# The Improvement Effects of a Nutritional Fortifier on the Reproductive Performance, Sex Steroid Hormone Production, and Health of the Striped Bamboo Shark *Chiloscyllium plagiosum*

**DOI:** 10.3390/ani14142112

**Published:** 2024-07-19

**Authors:** Junjie Zhang, Chao Xu, Yifan Zhang, Yifu Zhong, Dizhi Xie, Peng Zhang, Yuanyou Li

**Affiliations:** 1 College of Marine Sciences, South China Agricultural University, Guangzhou 510642, Chinaxiedizhi@scau.edu.cn (D.X.); 2 Guangdong Chimelong Group Co., Ltd., Guangzhou 511430, China

**Keywords:** *Chiloscylium plagiosum*, nutritional fortifier, reproductive performance, sex steroid hormone

## Abstract

**Simple Summary:**

Sharks belong to the class Chondrichthyes in the phylum Chordata. They are widely distributed in tropical, temperate, and polar oceans worldwide. As the top predator in the marine food chain, sharks have late sexual maturity and a long reproductive cycle. In recent years, due to the threat of illegal fishing and overfishing, their numbers have fallen sharply, and 23 species of sharks have been listed in protected lists, which poses a direct threat to the balance of marine ecosystems. In order to explore ways to increase the number of sharks by improving their reproductive performance under artificial breeding conditions, we used the striped bamboo shark (*Chilosophy plagiosum*) as an experimental animal, which has been listed as a near-threatened species in the Red List of Endangered Species of the World Conservation Union and legally bred in Chimelong Ocean Kingdom of Zhuhai, China. The results of the breeding experiment showed that feeding sharks a nutritional fortifier in the breeding season can improve the spawning quantity and egg quality of the striped bamboo shark, which provides new ideas and strategies for improving the protection and utilization level of shark resources, and has important economic, ecological, and social benefits.

**Abstract:**

To explore a method of improving the reproductive performance of the striped bamboo shark, three groups (D0, D1, and D2) of mature individuals were fed squid with (D1 and D2) or without (D0) a nutritional fortifier during the breeding seasons of 2022 and 2023. Compared with the D0 group, the D1 and D2 groups had an increase of 20.90% and 31.34% in total eggs, increases of 32.73% and 41.82% in the proportion of lecithal eggs, and a total 119.07% increase in hatching rate, respectively, in 2022. In 2023, the corresponding increase was 17.12% and 9.91% in total eggs, 19.63% and 12.15% in the proportion of lecithal eggs, 43.37% and 43.94% in fertilization rate, 23.94% and 22.22% in hatchability rate, and 66.70% and 8.70% in the survival rate of fry. Moreover, the levels of serum estradiol, testosterone, progesterone, albumin, and total antioxidant capacity and the levels of ARA, EPA, DHA, n-3 PUFA, and n-6 PUFA in both serum and lecithal eggs significantly increased, while the levels of serum triglyceride and total cholesterol were the opposite (*p* < 0.05). The results demonstrate that feeding the sharks with a nutritional fortifier can increase spawn production and the quality of eggs, regulate the production of sex steroids, and improve the nutrition of eggs and the health of broodstocks.

## 1. Introduction

The development of gonads in fish has a significant impact on reproductive performance, such as egg production, egg quality, and the survival rate of larvae. For example, a close correlation was observed in white mullet (*Mugil curema*) between the maturation of gonads and egg production [1]. Females with well-developed gonads tend to produce more and higher-quality eggs and sperm. Poorly developed gonads usually result in a decrease in the quality of sperm and eggs, affecting the fertilization rate and the subsequent development of larvae.

The nutritional quality of food is one of the key factors affecting the reproductive performance of fish. Fatty acids have been identified as important nutrients that affect reproductive processes through various mechanisms. For example, arachidonic acid (ARA) and n-6 polyunsaturated fatty acids (n-6 PUFA) can participate in follicle maturation and steroid production [2]. In addition, n-3 long chain PUFA (n-3 LC-PUFA) such as docosahexaenoic acid (DHA), eicosapentaenoic acid (EPA), and n-6 LC-PUFA like ARA, can affect fish reproduction by improving immune ability [3]. Both DHA and EPA are major components of cell membranes, including sperm and oocytes, and play an important role in the fertilization process [4]. However, due to the poor ability to synthesize DHA and EPA in marine teleost [5,6], the deficiency of these fatty acids is the significant cause that leads to poor gonadal development, decreased gamete quality, poor larval development, and high mortality rates in artificial fish juvenile cultivation [7,8,9].

The hypothalamic–pituitary–gonadal axis (HPG axis) plays a crucial role in influencing the reproductive performance of fish. The gonadal development process of fish is regulated by endocrine mechanisms within their reproductive system, which are mainly dominated by the HPG axis [10]. In this biological process, the hypothalamus secretes and releases gonadotropin-releasing hormones (GnRH), which can stimulate the pituitary gland to synthesize gonadotropins into the bloodstream, thereby promoting the production of sex hormones [11]. This endocrine regulation process is the core of gonadal development and function, ensuring the normal sexual maturation and reproduction of fish. Understanding this mechanism is of great significance for fish reproduction and conservation, as it can provide guidance for optimizing reproductive management.

In addition to the nutritional quality of food and the HPG axis, the healthy status of broodstocks is also one of the most important factors affecting the reproductive performance of animals. On this point, no information is available on the blood indicators used to assess the health of sharks. Studies in teleost fish have shown that serum physiological and biochemical indexes such as total antioxidant capacity (T-AOC), albumin (ALB), total cholesterol (TC), triglycerides (TG), and LC-PUFA are important indicators for detecting health and metabolism status [12,13,14]. They provide clues for this study to evaluate the influence of broodstocks’ health on reproductive performance by measuring shark blood parameters.

Sharks belong to the class Chondrichthyes in the phylum Chordata. They are widely distributed in tropical, temperate, and polar oceans worldwide. Sharks exhibit keen senses of smell, hearing, and touch. As carnivorous animals, sharks primarily eat small fish, sea turtles, seabirds, seals, and other marine creatures. As apex predators in the marine food chain, sharks have late sexual maturity and a long reproductive cycle. A significant decline in their numbers poses a direct threat to the balance of the marine ecosystem [15]. In recent years, sharks have faced threats from illegal fishing and overfishing, leading to 23 shark species being included in protected lists. The striped bamboo shark *Chiloscylium plagiosum* belongs to the family Scorpaenidae and has been listed as a near-threatened species on the IUCN Red List of Threatened Species. Therefore, the protection of its resources is urgent. Chimelong Group in China is a company dedicated to global wildlife conservation, breeding, and science education. It aims to fortify the efficiency of wildlife conservation efforts, raise public awareness about wildlife protection, and establish the Chimelong Animal and Plant Conservation Foundation in Guangdong Province of China to support the national strategy for the conservation of rare wildlife. The Chimelong Animal and Plant Research and Innovation Institute, through technological innovation and visionary leadership, is driving advancements in global animal and plant conservation. There are some *C. plagiosum* at the Chimelong Ocean Aquarium in Zhuhai, China, but their breeding numbers have been declining in recent years. In order to solve this problem, the present study aimed to pay attention to the nutrition of broodstocks, and thus, a nutritional fortifier was designed according to the nutritional requirements of broodstocks of some marine teleost due to the absence of corresponding data in sharks. The nutritional fortifier consisting of PUFA, vitamins, and amino acids was fed to the broodstocks of *C. plagiosum* during the breeding season with the aim of promoting the development and maturation of gonads and increasing reproductive performance, so as to provide new ideas and strategies for improving the protection and utilization of sharks.

## 2. Materials and Methods

### 2.1. Ethical Statement

The experimental procedure was approved by the Ethics of the Institutional Animal Care and Use Committee (IACUC) of Laboratory Animals of South China Agricultural University.

### 2.2. Design and Preparation of the Nutritional Fortifier

Due to the lack of data on the nutritional requirements of shark broodstocks, the nutritional fortifier used in this study was designed according to the nutritional requirements of broodstocks of bony fish [16,17,18], which consisted of 2.00% EPA, 4.00% DHA, 2.50% taurine, 0.05% vitamin E, 0.05% vitamin A, 0.02% antioxidant (butylated hydroxytoluene), and 91.38% wheat flour. After the first six ingredients were fully mixed with about one-tenth of the total wheat flour, the mixture was then mixed with the remaining wheat flour, and then some water was used to form a dough, which was then processed into bar-shaped strips of about 3 cm in length using an extruder. They were coated with dry flour and stored in a refrigerator at −20 °C for later use.

### 2.3. Animals and Experimental Design

The striped bamboo sharks were provided by the Chimelong Ocean Kingdom in Zhuhai, which belongs to Guangdong Chimelong Group Co., Ltd., Guangzhou 511430, China. The feeding experiment was conducted in the pool of the Whale Shark Pavilion at Chimelong Ocean Kingdom in Zhuhai, in the summer–autumn seasons of 2022 and 2023, respectively. The main purpose of the 2023 experiment was to further verify the positive findings obtained in 2022.

Three groups (D0, D1, and D2) of striped bamboo sharks were used for the feeding experiments each year. Individuals (approximately 2–2.5 kg) who came from the same group and had been sexually mature for several years were randomly assigned to each group at the beginning of the feeding experiment. The difference between the 2022 and 2023 experiments was that the ratio of males to females was slightly different in each group, i.e., 10 females and 6 males in 2022 and 8 females and 8 males in 2023. During the feeding experiment, three groups of sharks were cultured in the same flat-bottomed cement pool and closely separated by fishing nets fixed on the rectangular wooden frame so as to avoid the transfer of sharks between groups but shared the same water conditions. The males and females were cohabitated in the D0 and D1 groups. In order to know whether the sexual separation of sharks would affect their reproductive performance, the males and females in the D2 group were kept separate by a net in the initial stage of the experiment and then mixed together when noticeable signs of estrus were observed in the D0 or D1 group.

**Figure 1:**
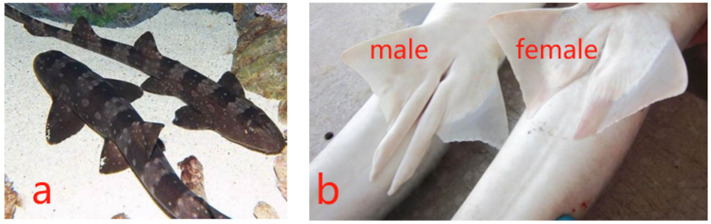
Photographs of striped bamboo sharks: (**a**) shark morphology and (**b**) the distinction between males and females.

Leveraging the striped bamboo sharks’ ability to swallow whole squid, the bar-shaped nutritional fortifier strips were inserted into the mantle of squids to minimize losses. Every day, the D0 group (control) was fed 400 g of squid only (25 g for each shark on average), while groups D1 and D2 were fed 400 g of squid supplemented with 48 g of the nutritional fortifier (3 g for each shark on average), fed every day at 10 a.m. The feeding experiment lasted 69 days from 2 August to 9 October 2022 and 87 days from 1 July to 25 September 2023. In the pool, the water temperature was 25.5–26 °C, the salinity was 33–34%, the pH was 7.6–8.4, the dissolved oxygen concentration was 6.5–9.5, and the ammonia nitrogen concentration was ≤0.01. Photographs of the sharks are shown in Figure 1.

### 2.4. Sample Collection

Blood samples were collected from the caudal vein of each shark on 1 August, 8 September, and 10 October 2022 and on 30 June, 30 July, 29 August, and 24 September 2023. After centrifugation at 3000 × *g* for 10 min (4 °C), serums were collected and kept at −70 °C until analysis.

A flashlight was used to distinguish lecithal eggs (containing lots of yolk) and alecithal eggs (containing little yolk). The collected lecithal eggs were put in an incubator for hatching, and three batches of lecithal eggs were collected for analysis of proximate and fatty acid composition.

### 2.5. Determination of Serum Indices

Serum estradiol, testosterone, and progesterone levels were measured via an enzyme-linked immunoabsorbent method using a zebrafish (*Danio rerio*) antibody as the standard (Shanghai Enzyme-linked Biotechnology Co., Ltd., Shanghai, China). Serum physiological biochemical indices (superoxide dismutase, catalase, malondialdehyde, total antioxidant capacity, triglycerides, and total cholesterol) were measured using reagent kits from Nanjing Jiancheng Bioengineering Institute (Nanjing, China).

### 2.6. Determination of Proximate Composition and Fatty Acids of Lecithal Eggs

Determination of the proximate composition of lecithal eggs was performed according to the standard methods of the Association of Official Analytical Chemists. Dry matter was determined by constant weight after drying at 10 °C; crude protein (N × 6.25) was measured by using the Kjeldahl method with a semi-automatic Kjeldahl apparatus (KDN-102C, Shanghai Xianjian Instruments, Shanghai, China) following acid digestion; crude lipid content was determined via Soxhlet extraction (ST 255, Soxtec, Foss, Suzhou, China); and ash was carbonized in an electric ceramic furnace and then burned in a muffle furnace at 550 °C for 4 h. The fatty acid composition of the lipids extracted from the lecithal eggs and serum was determined using a gas chromatograph (7890B; Agilent Technologies, Santa Clara, CA, USA) as used previously [19].

### 2.7. Calculation Formulas

Calculation formulas are shown below:

Proportion of residual lecithal eggs (%) = Residual lecithal eggs/Total egg production × 100

Fertility rate (%) = Number of fertilized eggs/Total egg production × 100

Hatchability rate (%) = Number of larvae hatched/Number of fertilized eggs × 100

Survival rate of juveniles (%) = Number of surviving juveniles/Number of larvae hatched × 100

### 2.8. Data Statistical Analysis

The data for all analyses are expressed as the means ± SEM. All data were analyzed via one-way ANOVA with SPSS software (Version 22.0, Chicago, IL, USA) for Windows. Tukey’s multiple comparisons test was used to analyze the significance of differences among the groups, and *p* < 0.05 was considered as significant.

## 3. Results

### 3.1. Reproductive Performance

In the feeding experiments of 2022 and 2023, egg laying began on 21 August and 20 July, and the experiments ended on 9 October and 25 September, respectively. The number of eggs laid by the striped bamboo sharks in 48 days in 2022 and 67 days in 2023 among the different experimental groups is shown in Table 1. The results show that the number of lecithal eggs was 55, 73, and 78, and total egg production was 67, 81, and 88 in groups D0, D1, and D2, respectively, in 2022. Compared to group D0, the proportion of lecithal eggs in groups D1 and D2 increased by 32.73% and 41.82%, and total egg production increased by 20.90% and 31.34%, respectively. In 2023, the number of lecithal eggs was 107, 128, and 120, and total egg production was 111, 130, and 122, respectively, in groups D0, D1, and D2. Compared to group D0, the D1 and D2 groups obtained increases of 19.63% and 12.15% in the proportion of lecithal eggs, respectively, and increases of 17.12% and 9.91% in total eggs. The results over two consecutive years demonstrate that the nutritional fortifier significantly increased the number of eggs laid by the sharks.

In 2022, 87 lecithal eggs from the D1 and D2 groups and 81 lecithal eggs from the D0 group were used to compare the hatching effect, and 40 and 17 larvae hatched, with hatching rates of 45.98% and 20.99%, respectively, and the former is 119.07% higher than the latter. In 2023, the quality of eggs was comprehensively evaluated, as shown in Table 2. The results indicate that, compared with group D0, groups D1 and D2 respectively displayed increases of 23.81% and 21.31% in the proportion of residual lecithal eggs, increases of 43.37% and 43.94% in their fertilization rate, increases of 23.94% and 22.22% in hatching rates, and increases of 66.70% and 8.70% in the survival rate of fry. These results indicate that groups D1 and D2 showed obvious increases in total egg production, fertilization rates, hatchability rate, and the survival rate of fry, demonstrating that feeding the sharks with a nutritional fortifier can effectively improve the quantity and quality of eggs.

### 3.2. Biochemical Composition of Eggs and Fatty Acid Composition in Serum and Eggs

The proximate composition of lecithal eggs produced in 2022 and 2023 is shown in Table 3. The results showed that the content of crude protein significantly increased, while that of moisture significantly decreased (*p <* 0.05) in the D1 and D2 groups compared with those of the D0 group. The results indicate that the nutritional fortifier can increase the protein content of eggs.

The fatty acid composition of serum and lecithal eggs from striped bamboo shark broodstocks in 2022 and 2023 are shown in Table 4 and Table 5, respectively. Compared with those of the D0 group, the D1 and D2 groups exhibited a significantly higher level of ARA, EPA, DHA, n-6 PUFA, and n-3 PUFA in their serum, as well as higher levels of 16:00 and 18:00, DHA, n-6 PUFA, and n-3 PUFA in lecithal eggs (*p <* 0.05). These results indicate that the nutritional fortifier can improve the nutrition of eggs.

### 3.3. Serum Physiological and Biochemical Indicators

**Figure 2:**
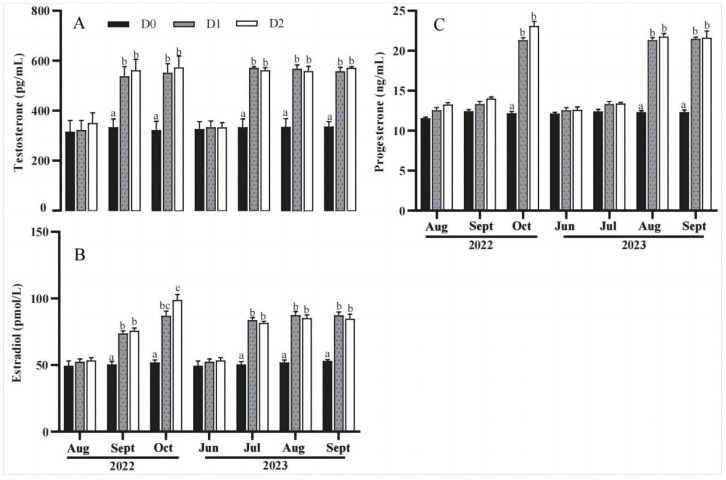
The levels of serum testosterone (**A**), estradiol (**B**) and progesterone (**C**) of *Chiloscyllium plagiosum* in the different experimental groups during 2022 or 2023. The values are presented as the mean ± SEM (*n* = 3). Bars assigned different superscripts were significantly different (*p* < 0.05).

The levels of serum steroid hormones are shown in Figure 2. The results showed that their levels had no significant difference (*p* > 0.05) among the D0, D1, and D2 groups before the start of the feeding experiment (August 2022, June 2023). After feeding the sharks in the D1 and D2 groups with the nutritional fortifier, the serum levels of testosterone and estradiol increased earlier than those of progesterone, i.e., the levels of testosterone and estradiol were significantly higher than those of the D0 group in the first and following months (September and October 2022; July, August, and September 2023) (Figure 2A,B), while progesterone displayed significantly higher levels only after feeding the sharks on the nutritional fortifier for two months (Figure 2C) (*p <* 0.05). The results indicate that the nutritional fortifier can increase the levels of serum testosterone, estradiol, and progesterone in sharks.

**Figure 3:**
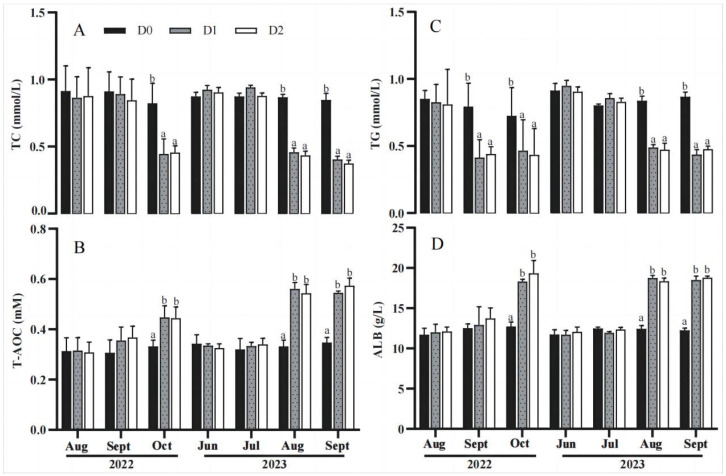
Serum biochemical indexes of *Chiloscyllium plagiosum* in the different experimental groups during 2022 or 2023. The values are presented as the mean ± SEM (*n* = 3). Bars assigned with different superscripts were significantly different (*p* < 0.05). (**A**): triglyceride (TC); (**B**): total antioxidant capacity (T-AOC); (**C**): total cholesterol (TC); (**D**): albumin (ALB).

Serum biochemical indexes are shown in Figure 3. The results showed that all of the indexes including the total antioxidant capacity (T-AOC), albumin (ALB), total cholesterol (TC), and triglycerides (TG) had no differences among the D0, D1, and D2 groups before the start of the feeding experiment (August 2022 and June 2023). After feeding the sharks in the D1 and D2 groups with the nutritional fortifier for one or two months, T-AOC and ALB content significantly increased (*p <* 0.05) (Figure 3B,D), while the levels of TC and TG significantly decreased (*p <* 0.05) (Figure 3A,C) compared with the D0 group. The results indicate that the nutritional fortifier can improve the serum lipid metabolism, antioxidant capacity, and immunity of bamboo sharks.

## 4. Discussion

### 4.1. A Nutritional Fortifier Can Obviously Increase the Reproductive Performance of the Striped Bamboo Shark

The number of eggs laid in 2022 and 2023 as shown in Table 1 demonstrates that feeding the striped bamboo shark with nutritional fortifier obviously increases their spawning production. As to the difference in the number of eggs laid and the increase rate of lecithal eggs (%) or total eggs (%) between 2022 and 2023, one of the possible reasons may be due to the difference in spawning time and the number of days to collect eggs, which is from 21 August to 9 October and 48 days in 2022 and from 20 July to 25 September and 67 days in 2023. Moreover, this is the first time for us to use sharks to conduct a reproduction experiment, which resulted in the absence of comprehensively evaluating the quality of eggs in 2022. Encouragingly, the results in 2023 demonstrate that feeding the striped bamboo shark with a nutritional fortifier also obviously improves the quality of eggs as evaluated by the indexes in the proportion of residual lecithal eggs, fertilization rate, hatching rates, and survival rate of fry, as shown in Table 2. These encouraging results caused us to explore its mechanism.

### 4.2. Possible Mechanisms of the Improvement Effects of a Nutritional Fortifier on the Reproductive Performance of the Striped Bamboo Shark

#### 4.2.1. A Nutritional Fortifier May Increase the Reproductive Performance of Striped Bamboo Sharks by Improving the Nutritional Composition of Eggs

Generally, there is a relationship between the reproductive performance of broodstocks and dietary nutrition. The nutritional composition of eggs can reflect the physiological health and nutritional status of broodstock during spawning, thus playing an extremely important role in aquatic animal reproduction. Fish ingest nutrients from external food and then undergo a series of digestion and absorption processes to convert these substances into their own metabolisms [20]. In the present research, the nutritional fortifier contained PUFA, vitamins, and amino acids. In the meantime, the contents of crude protein and crude lipids and the levels of ARA, EPA, DHA, n-6 PUFA, and n-3 PUFA in eggs of the D1 and D2 groups were significantly higher than those of the D0 group, while the water content was the opposite (*p* < 0.05). These results suggest that the improvement of egg production and quality including the proportion of lecithal eggs, fertilization rate, hatching rate, and survival rate of fry in D1 and D2 groups may be due to, to a certain degree, the improvement of the nutritional composition of eggs by feeding the broodstocks with a nutritional fortifier. These results are similar to our earlier report in broodstocks of marine teleost *Plectorhynchus cinctus*, which showed that feeding fish a diet with 1.27% to 2.36% n-3 LC-PUFA resulted in improved reproductive performance such as egg production, fertilization rate, the survival rate of juveniles, and the body length of juveniles [19]. The present results of two consecutive years indicate that the nutritional fortifier can significantly increase the spawning quantity of sharks. However, there was a difference in the proportion of lecithal eggs and the total egg production between 2022 and 2023; the potential reasons may be attributed to variations in the sex ratio and the status of the shark population. In the 2023 experiment, the sex ratio between females and males was 1:1, whereas there were relatively more females in the 2022 experiment. The difference in sex ratio may impact the competition and coordination levels between them, affecting their reproductive behavior and consequently affecting spawning quantity and quality.

In addition, a difference existed between the D1 and D2 groups in the proportion of lecithal eggs and the total egg production in the 2022 experiment, which suggests that the separate rearing of females and males in the initial stage can increase reproductive performance as seen in Group D2. However, such results were not confirmed in the 2023 experiment. We are not sure whether this was due to the apparent difference in sex ratio between 2023 and 2022. This needs to be further studied and confirmed.

#### 4.2.2. A Nutritional Fortifier May Increase the Reproductive Performance of Striped Bamboo Sharks by Regulating Steroid Hormone Production

Sex steroid hormones play a crucial role in the reproductive ecology of sharks [21]. Testosterone (T) is an important male steroid hormone that plays a crucial role in the reproductive system of male fish by promoting the production and maturation of sperm, as well as affecting the behavior and color characteristics of male fish [22]. In this study, feeding the sharks with a nutritional fortifier increased serum testosterone levels; this may be one of the reasons for improving the fertilization rate by increasing the quality and quantity of sperm. Estradiol (E_2_) is the main representative of female steroid hormones, which affect the development and function of the reproductive system of female fish. Estradiol can promote the growth and development of ovarian follicles, as well as regulate the reproductive behavior of broodstock [23]. In this study, the nutritional fortifier increased the secretion of estradiol, thereby accelerating egg development. Progesterone (P), as a luteal hormone, plays a crucial role in pregnancy and embryonic development. It can regulate some hormones secreted by the pituitary gland, thereby affecting the growth and function of reproductive organs. The nutritional fortifier increased the level of progesterone, which can maintain pregnancy status, thereby improving the hatching rate and litter survival rate [24]. Moreover, follicle-stimulating hormone and luteinizing hormone are part of the gonadotropins, which together promote the secretion of estrogen, thereby enhancing the estrogen effect of broodstock. Qiang (2021) found that the levels of E_2_ and progesterone increase after the ovarian development of Nile tilapia, which indicated that the maturation of ovaries is closely related to changes in the content of sex steroid hormones [25]. The level of estradiol in *Pampus argenteus* is positively correlated with the liver weight index (HSI), while the level of testosterone is significantly positively correlated with the gonadal maturity index (GSI) [26]. In this study, the increase in serum progesterone level appeared relatively later than that of testosterone and estradiol; this coincides with their difference in function. Our earlier study in marine teleost *P. cinctus* broodstocks reported that dietary n-3 LC-PUFA content can affect gonadal development together with testosterone and estradiol levels, thereby improving egg production as well as egg and larval quality [27]. In addition, Xu (2017) found that DHA:EPA ratios can affect serum testosterone levels and improve the reproductive performance of flounder [28]. In this study, one of the main constituents in the nutritional fortifier was LC-PUFA HUFA. This suggests that the enhancing effects on sex steroid hormone production may be one of the mechanisms by which the nutritional fortifier improved the reproductive performance of the striped bamboo sharks.

#### 4.2.3. A Nutritional Fortifier May Increase Reproductive Performance by Improving the Health of the Striped Bamboo Shark

Serum components can reflect the physiological health of fish [29]. The organism’s antioxidant enzyme defense system plays a vital role in clearing or neutralizing oxygen radicals in the body, helping to maintain the relative stability of the biological system [30]. For example, superoxide dismutase is an enzyme that catalyzes the dismutation of superoxide anions (O_2_^−^) into hydrogen peroxide and oxygen. It is widely distributed in various organisms, and its activity is closely related to the degree of cellular damage. A lack of antioxidant enzymes or decreased activity can lead to an imbalance in free radical metabolism in the body, thereby adversely affecting cellular substance exchange and energy metabolism [12]. Therefore, understanding these serum physiological and biochemical indicators is crucial for assessing the physiological status and health of fish. Total antioxidant capacity (T-AOC) is an indicator of the overall antioxidant level of the organism, encompassing various antioxidants and antioxidant enzymes. Therefore, T-AOC is an important indicator for evaluating the organism’s antioxidant capacity. Huang et al. (2016) found that LC-PUFA significantly increased the total antioxidant capacity of grass carp [14]. In this study, the total T-AOC and ALB content in the D1 and D2 groups were significantly higher than those in the D0 group (*p* < 0.05) (Figure 3B,D).

Serum lipids, such as triglycerides, can reflect the status of lipid metabolism in the body [31]. In this study, sharks in the D1 and D2 groups fed the nutritional fortifier displayed notably reduced levels of triglycerides and total cholesterol compared to those in the D0 (control) group. These results suggest a positive influence of the nutrient fortifier on lipid metabolism and cholesterol transport in sharks, potentially due to the presence of n-3 LC-PUFA in the nutritional fortifier, which plays a pivotal role in regulating lipid metabolism and cholesterol transport by impacting processes like fatty acid synthesis and degradation. These fatty acids aid in modifying the fatty acid composition, resulting in healthier fatty acid profiles characterized by lower saturated fatty acid content and higher unsaturated fatty acid content through the modulation of cell membrane structure and function. Other studies have shown that n-3 LC-PUFA can improve growth performance, disease resistance, and environmental adaptation capabilities in rats [32] and enhance the fatty acid profiles, lipid metabolism, and antioxidant capacity in the golden pompano *Trachinotus ovatus* [33]. All of the above results suggest that the improvement effects on health indexes such as serum T-AOC, ALB, triglycerides, and total cholesterol may be one of the mechanisms by which the nutritional fortifier improved the reproductive performance of striped bamboo sharks.

## 5. Conclusions

The present study indicates that feeding the broodstocks of striped bamboo sharks with a nutritional fortifier can obviously increase egg production and quality, which may be achieved by regulating the production of sex hormones, improving the nutrition of eggs and the health of broodstocks, and enhancing the development of gonads. This study is the first to explore the strategy of using nutritional fortifiers to enhance shark reproduction, which not only provides a new way to increase the number and protection level of striped bamboo sharks but also lays a scientific foundation for the application of nutritional fortifiers in other sharks.

## Figures and Tables

**Table 1:** Egg production of the striped bamboo shark *Chiloscyllium plagiosum* in 2022 and 2023 among the different experimental groups.

Items	2022			2023		
	D0	D1	D2	D0	D1	D2
Lecithal ggs	55	73	78	107	128	120
Alecithal eggs	12	8	10	4	2	2
Total eggs	67	81	88	111	130	122
Increase in lecithal eggs (%)	/	32.73	41.82	/	19.63	12.15
Increase in total eggs (%)	/	20.90	31.34	/	17.12	9.91

Notes: D0—sharks fed squid without providing a nutritional fortifier, D1—sharks fed squid with a nutritional fortifier, and the females and males were mixed; D2—sharks fed squid with a nutritional fortifier but the females and males were separated during the initial stage of the experiment. Lecithal egg: an egg with lots of yolk; alecithal egg: an egg with little yolk. The notes in the other tables are the same.

**Table 2:** Quality of eggs of the striped bamboo shark *Chiloscyllium plagiosum* produced in 2023 among the different experimental groups.

Items	D0	D1	D2
Total eggs	111	130	122
Residual lecithal eggs	60	87	80
Proportion of residual lecithal eggs (%)	54.05	66.92	65.57
Increase in the proportion of residual lecithal eggs (%)	/	23.81	21.31
Fertilized eggs	31	52	49
Fertility rate (%)	27.92	40.00	40.16
Increase in fertility rate (%)	/	43.37	43.94
Hatchability rate (%)	54.55	67.61	66.67
Increase in hatchability rate (%)	/	23.94	22.22
Survival rate of fry (%)	10.00	16.67	10.87
Increase in the survival rate of fry (%)	/	66.70	8.70

Notes: The same as Table 1. Proportion of residual lecithal eggs (%) = Residual lecithal eggs/Total egg production × 100. Fertility Rate (%) = Number of fertilized eggs/Total Egg × 100. Hatchability rate (%) = Number of larvae hatched/Number of fertilized eggs × 100. Survival rate of fry (%) = Number of surviving fry/Number of larvae hatched × 100.

**Table 3:** The proximate composition of *Chiloscyllium plagiosum* lecithal eggs produced in 2022 and 2023 among the different experimental groups.

Items		2022			2023	
	D0	D1	D2	D0	D1	D2
Dry matter	83.53 ± 0.35 ^b^	82.72 ± 0.28 ^a^	83.53 ± 0.35 ^b^	80.06 ± 0.11 ^b^	77.64 ± 0.11 ^a^	77.62 ± 0.12 ^a^
Crude protein	18.89 ± 0.10 ^a^	19.41 ± 0.26 ^b^	18.89 ± 0.10 ^a^	15.15 ± 0.05 ^a^	18.24 ± 0.04 ^b^	18.16 ± 0.05 ^b^
Crude lipid	5.62 ± 0.22 ^a^	6.51 ± 0.21 ^b^	5.62 ± 0.22 ^a^	3.24 ± 0.07	3.26 ± 0.06	3.14 ± 0.06
Ash	0.93 ± 0.12	0.80 ± 0.18	0.93 ± 0.12	0.83 ± 0.01	0.81 ± 0.01	0.83 ± 0.02

Note: The values are the means ± SEM of 3 replications (*n* = 3). Values in 2022 or 2023 within a row not sharing a common superscript letter indicate significant differences (*p* < 0.05), while those with no superscript letters indicate no significant difference (*p* > 0.05) according to Tukey’s multiple comparisons test.

**Table 4:** Fatty acid composition of serum from striped bamboo shark *Chiloscyllium plagiosum* broodstocks in 2022 and 2023 among the different experimental groups.

Fatty Acids (Total %)		2022			2023	
	D0	D1	D2	D0	D1	D2
12:0	1.33 ± 0.05	1.49 ± 0.14	1.71 ± 0.09	1.52 ± 0.02	1.45 ± 0.11	1.58 ± 0.05
14:0	4.96 ± 0.08	5.33 ± 0.76	4.58 ± 0.66	4.85 ± 0.12	4.73 ± 0.21	4.45 ± 0.23
16:0	27.73 ± 0.12	29.05 ± 0.18	30.1 ± 0.11	29.81 ± 0.22	29.11 ± 0.24	29.82± 0.22
18:0	6.75 ± 0.26	6.54 ± 0.71	6.46 ± 0.9	6.18 ± 0.19	6.74 ± 0.33	6.35 ± 0.41
SFA	41.58 ± 0.7	44.2 ± 1.03	44.61 ± 0.26	42.91 ± 0.24	42.92 ± 0.23	43.11 ± 0.16
16:1	2.58 ± 0.09	2.56 ± 0.11	2.52 ± 0.16	2.42 ± 0.04	2.39 ± 0.06	2.46 ± 0.08
18:1n-9	32.87 ± 0.79	32.81 ± 1.63	33.64 ± 0.68	33.07 ± 0.72	32.91 ± 0.63	33.41 ± 0.42
24:1n-9	0.89 ± 0.03	0.61 ± 0.06	0.91 ± 0.18	0.85 ± 0.06	0.91 ± 0.06	0.83 ± 0.08
MUFA	39.53 ± 0.7	41.42 ± 1.03	40.24 ± 0.26	39.92 ± 0.52	39.98 ± 0.61	40.04 ± 0.45
18:2n-6 (LA)	8.55 ± 0.37	9.44 ± 0.43	8.97 ± 0.85	9.05 ± 0.57	9.13 ± 0.87	8.89 ± 0.95
20:4n-6 (ARA)	2.11 ± 0.06 ^a^	4.68 ± 0.12 ^b^	4.85 ± 0.1 ^b^	2.03 ± 0.05 ^a^	4.45 ± 0.12 ^b^	4.32 ± 0.08 ^b^
n-6 PUFA	11.09 ± 0.06 ^a^	14.67 ± 0.22 ^b^	14.77 ± 0.05 ^b^	11.7 ± 0.05 ^a^	14.57 ± 0.09 ^b^	14.24 ± 0.05 ^b^
18:3n-3 (ALA)	3.34 ± 0.09	2.94 ± 0.22	2.96 ± 0.16	3.14 ± 0.05	3.22 ± 0.12	2.96 ± 0.11
20:5n-3 (EPA)	3.30 ± 0.01 ^a^	6.22 ± 0.09 ^b^	7.26 ± 0.11 ^b^	3.76 ± 0.03 ^a^	6.51 ± 0.08 ^b^	6.82 ± 0.06 ^b^
22:6n-3 (DHA)	4.45 ± 0.18 ^a^	6.53 ± 0.04 ^b^	6.78 ± 0.26 ^b^	4.33 ± 0.28 ^a^	6.86 ± 0.23 ^b^	6.97 ± 0.22 ^b^
n-3 PUFA	11.37 ± 0.2 ^a^	16.04 ± 0.24 ^b^	17.53 ± 0.37 ^b^	11.59 ± 0.16 ^a^	17.02 ± 0.22 ^b^	17.14 ± 0.16 ^b^
n-3/n-6	1.02 ± 0.06	1.09 ± 0.08	1.18 ± 0.07	0.99 ± 0.03	1.16 ± 0.05	1.20 ± 0.09

Note: The values are means ± SEM of 3 replications (*n* = 3). Values in 2022 or 2023 within a row not sharing a common superscript letter indicate significant differences (*p* < 0.05), while those with no superscript letters indicate no significant difference (*p* > 0.05) according to Tukey’s multiple comparisons test.

**Table 5:** Fatty acid composition of lecithal egg produced by the striped bamboo shark *Chiloscyllium plagiosum* in 2022 and 2023 among the different experimental groups.

Fatty Acids (Total %)		2022			2023	
	D0	D1	D2	D0	D1	D2
12:0	1.5 ± 0.05	1.49 ± 0.14	1.71 ± 0.09	1.55 ± 0.03	1.59 ± 0.11	1.62 ± 0.06
14:0	4.96 ± 0.08	5.33 ± 0.76	4.38 ± 0.66	4.86 ± 0.15	4.97 ± 0.11	4.82 ± 0.16
16:0	24.73 ± 0.2 ^a^	33.05 ± 0.88 ^b^	34.1 ± 0.59 ^b^	32.6 ± 0.17	33.42 ± 0.22	33.13 ± 0.23
18:0	6.75 ± 0.26 ^a^	8.84 ± 0.71 ^b^	9.06 ± 0.7 ^b^	8.85 ± 0.09	8.78 ± 0.17	9.10 ± 0.15
SFA	38.53 ± 0.7 ^a^	51.2 ± 1.03 ^b^	51.29 ± 0.26 ^b^	48.3 ± 0.33	49.37 ± 0.41	49.37 ± 0.22
16:1	2.58 ± 0.09	2.56 ± 0.11	2.52 ± 0.16	2.65 ± 0.09	2.51 ± 0.05	2.59 ± 0.06
18:1n-9	35.87 ± 0.79	31.81 ± 1.63	32.64 ± 0.68	33.42 ± 0.29	32.84 ± 0.22	32.92 ± 0.18
24:1n-9	0.89 ± 0.03	0.61 ± 0.06	0.91 ± 0.18	0.90 ± 0.05	0.85 ± 0.06	0.83 ± 0.08
MUFA	40.25 ± 0.84	36.78 ± 1.12	36.78 ± 0.39	36.78 ± 0.42	36.53 ± 0.72	36.53 ± 0.51
18:2n-6 (LA)	8.55 ± 0.37	8.44 ± 0.23	8.67 ± 0.25	8.23 ± 0.22	8.54 ± 0.33	8.17 ± 0.25
20:4n-6 (ARA)	0.51 ± 0.06 ^a^	0.88 ± 0.02 ^b^	0.86 ± 0.03 ^b^	0.55 ± 0.06 ^a^	0.89 ± 0.05 ^b^	0.86 ± 0.03 ^b^
n-6 PUFA	9.49 ± 0.06	9.57 ± 0.42	9.81 ± 0.05	9.06 ± 0.15	9.81 ± 0.12	9.31 ± 0.05
18:3n-3 (ALA)	3.04 ± 0.09	2.94 ± 0.22	2.76 ± 0.16	3.32 ± 0.05	3.02 ± 0.09	3.15 ± 0.11
20:5n-3 (EPA)	2.32 ± 0.01	2.17 ± 0.09	2.36 ± 0.11	2.36 ± 0.03	2.29 ± 0.09	2.31 ± 0.01
22:6n-3 (DHA)	3.15 ± 0.28 ^a^	5.03 ± 0.24 ^b^	5.14 ± 0.46 ^b^	3.21 ± 0.11 ^a^	5.63 ± 0.14 ^b^	5.45 ± 0.26 ^b^
n-3 PUFA	8.89 ± 0.2 ^a^	10.69 ± 0.24 ^b^	10.99 ± 0.37 ^b^	9.08 ± 0.22 ^a^	11.26 ± 0.24 ^b^	11.29 ± 0.15 ^b^
n-3/n-6	0.93 ± 0.06	1.11 ± 0.08	1.12 ± 0.07	1.00 ± 0.02	1.14 ± 0.05	1.21 ± 0.07

Note: The values are means ± SEM of 3 replications (*n* = 3). Values in 2022 or 2023 within a row not sharing a common superscript letter indicate significant differences (*p* < 0.05), while those with no superscript letters indicate no significant difference (*p* > 0.05) according to Tukey’s multiple comparisons test.

## Data Availability

All data are contained within the article.
